# Data supporting Ni-NTA magnetic bead-based fluorescent protease assay using recombinant fusion protein substrates

**DOI:** 10.1016/j.dib.2018.03.031

**Published:** 2018-03-12

**Authors:** János András Mótyán, Márió Miczi, Beáta Bozóki, József Tőzsér

**Affiliations:** a Department of Biochemistry and Molecular Biology, Faculty of Medicine, University of Debrecen, POB 6, H-4012 Debrecen, Hungary; b Biotechnological Analytical Department, Gedeon Richter Plc, 19-21. Gyömrői Rd, Budapest, H-1103 Hungary

**Keywords:** Recombinant fusion protein substrate, Protease assay, Fluorescent protein

## Abstract

Data provided here are related to the research article entitled as ‘A recombinant fusion protein-based, fluorescent protease assay for high throughput-compatible substrate screening’. Here we describe data related to the investigation of the properties of the His_6_-MBP-VSQNY↓PIVQ-mApple recombinant protein substrate and its interactions with Ni-NTA magnetic beads, including the dependence of substrate attachment on incubation time and concentration. Data on the folding efficiency and conformational stability of the recombinant substrate assessed by tryptic digestion are also presented. We describe here the changes of fluorescent properties and binding abilities upon treatments commonly used for stopping enzymatic reactions: trichloroacetic acid (TCA) or heat treatment.

**Specifications Table**

**Table:** 

**Subject area**	*Biology*
**More specific subject area**	*Biochemistry, Recombinant Proteins, Enzymatic assays and analysis*
**Type of data**	*Figure (SDS-PAGE image), graph*
**How data was acquired**	*SDS-PAGE, Fluorimetry (Biotek Synergy2 multimode plate reader), Densitometry*
**Data format**	*Analyzed*
**Experimental factors**	*Purified His*_*6*_*-MBP-VSQNY↓PIVQ-mApple recombinant fusion protein substrate in cleavage buffer (50 mM sodium-acetate, 300 mM NaCl, 0.05% Tween 20, pH 7.0)*
**Experimental features**	*Tryptic digestion and binding tests of untreated and treated His*_*6*_*-MBP-VSQNY↓PIVQ-mApple to Ni-NTA magnetic agarose beads*
**Data source location**	*not applicable*
**Data accessibility**	*Data is with this article.*

**Value of the data**

• Data from tryptic digestion may provide useful information on the folding properties of the fluorescent substrates
• In the case of homogenous protease assays, solution phase extraction may be used to remove substrates and N-terminal cleavage fragments from reaction mixture, the dependence of binding efficiency on the concentration of the applied magnetic beads and the incubation time need to be optimized for efficient attachment
• Exploring the changes in the binding abilities and fluorescent properties of His_6_-MBP-VSQNY↓PIVQ-mApple recombinant protein upon TCA- treatment or heat-denaturation may provide valuable data for the methods used to stop enzyme reactions in the case of a homogenous assay protocol

## 1 Data

**Fig. 1:**
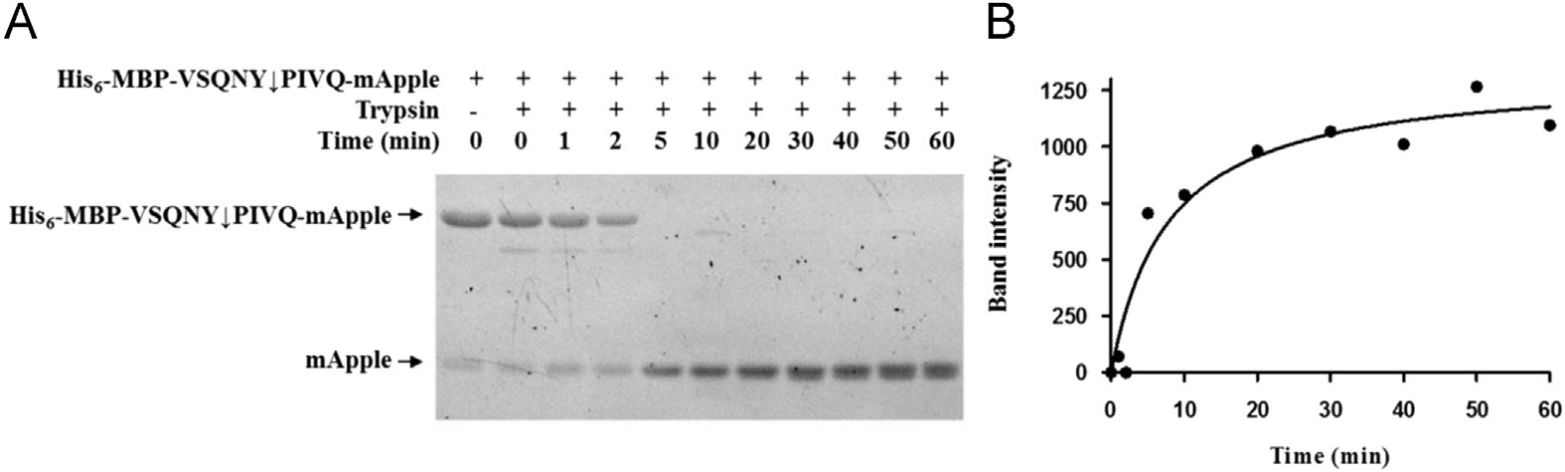
Digestion of His_6_-MBP-VSQNY↓PIVQ-mApple substrate by trypsin. The recombinant His_6_-MBP-VSQNY↓PIVQ-mApple substrate was incubated with trypsin up to 60 minutes. Samples were analyzed by SDS-PAGE, and the full-length His_6_-MBP-VSQNY↓PIVQ-mApple substrate and mApple fluorescent protein-containing C-terminal fragments were detected by UV imaging (**A**). The band intensities for mApple were plotted as a function of incubation time (**B**).

**Fig. 2:**
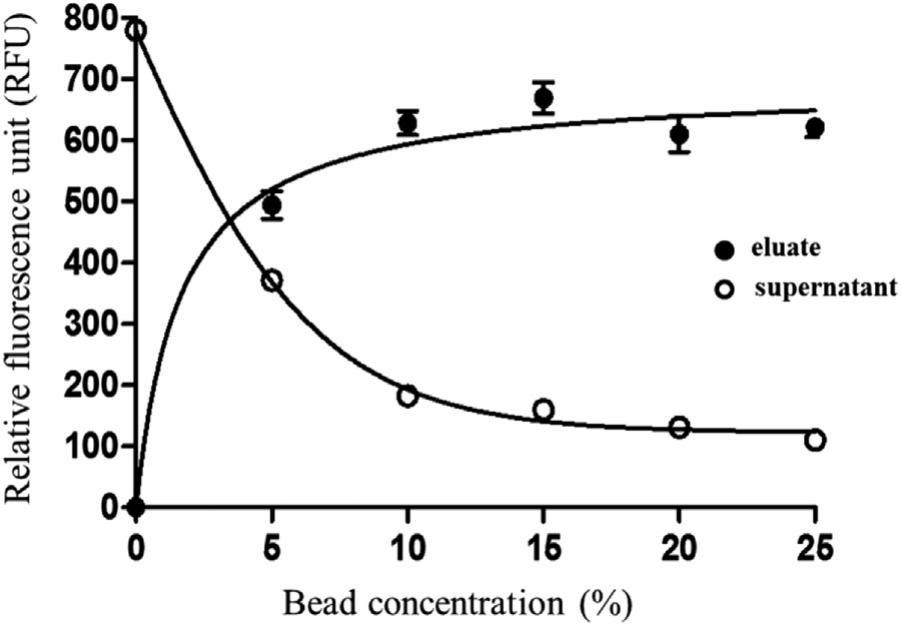
Dependence of substrate binding to the Ni-NTA coated magnetic beads on bead concentration. The dependence of His_6_-MBP-VSQNY↓PIVQ-mApple recombinant substrate attachment to Ni-NTA coated magnetic beads on bead concentration is shown in the graph. Magnetic bead suspensions were set to different concentrations and were incubated with constant amount of purified substrate. Fluorescence of both supernatant and eluate fractions were measured and plotted against bead concentration. Eluate fractions contain the bead-attached molecules, while supernatant fractions contain the molecules remained in the solution. Fluorescent backgrounds of the supernatant fraction samples are caused by the presence of small amount of free mApple in the substrate solution. Error bars represent SD (n = 2).

**Fig. 3:**
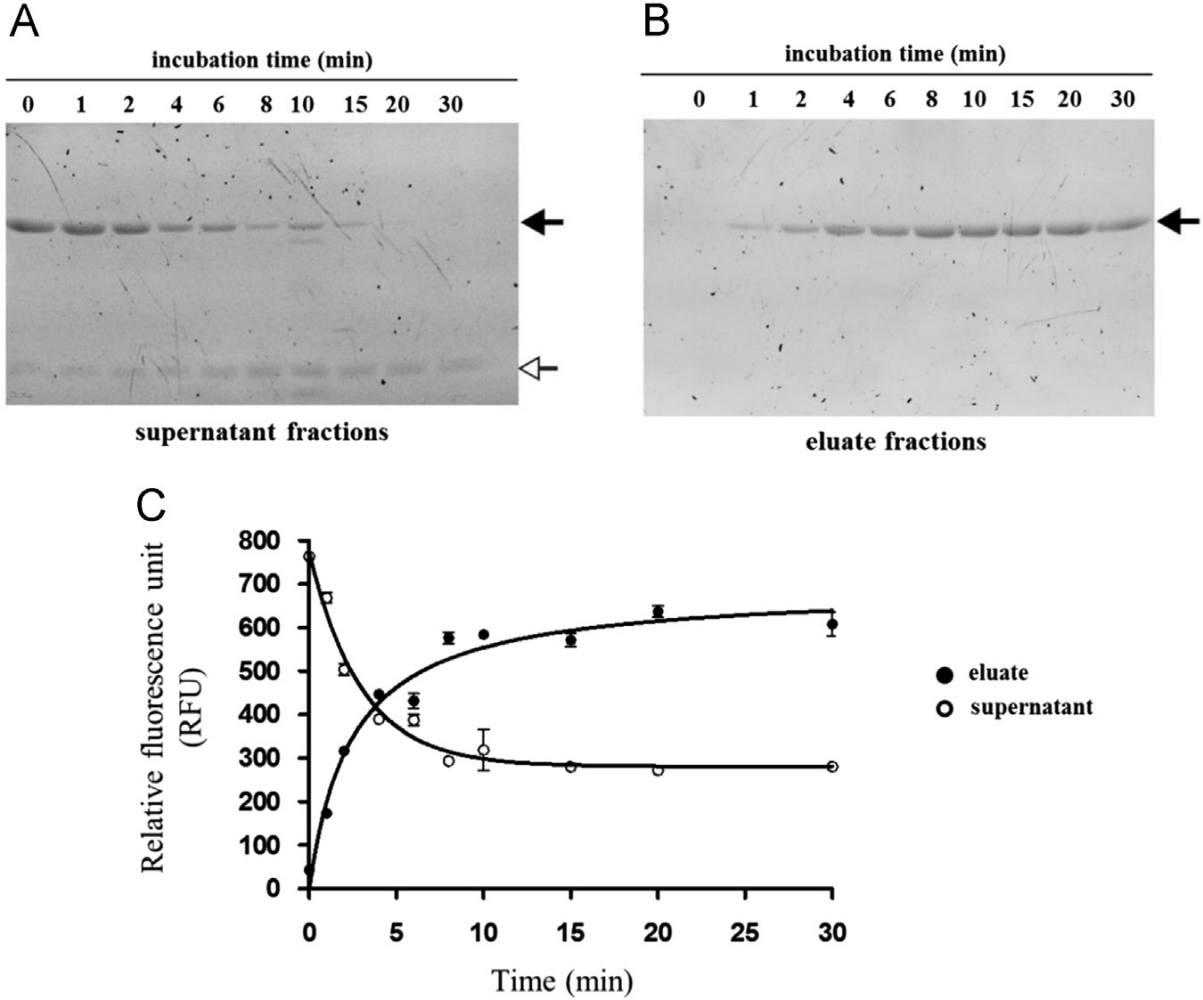
Dependence of substrate binding to the Ni-NTA coated magnetic beads on incubation time. The dependence of His_6_-MBP-VSQNY↓PIVQ-mApple recombinant substrate attachment to Ni-NTA coated magnetic beads on incubation time is represented. Magnetic bead concentration was set to be constant, while the time of incubation was changed from 0 to 30 minutes. Eluate fractions contain the bead-attached molecules, while supernatant fractions contain the molecules remained in the solution. Supernatant and eluate fractions were analyzed by denaturing SDS-PAGE, and both full-length His_6_-MBP-VSQNY↓PIVQ-mApple substrate (shown by black arrow) and mApple fluorescent protein (white arrow) were detected by UV imaging (**A and B**). Fluorescence of supernatant and eluate fractions were measured and plotted against incubation time (**C**). Fluorescent backgrounds of the supernatant fraction samples are caused by the presence of small amount of free mApple in the substrate solution. Error bars represent SD (n = 2).

**Fig. 4:**
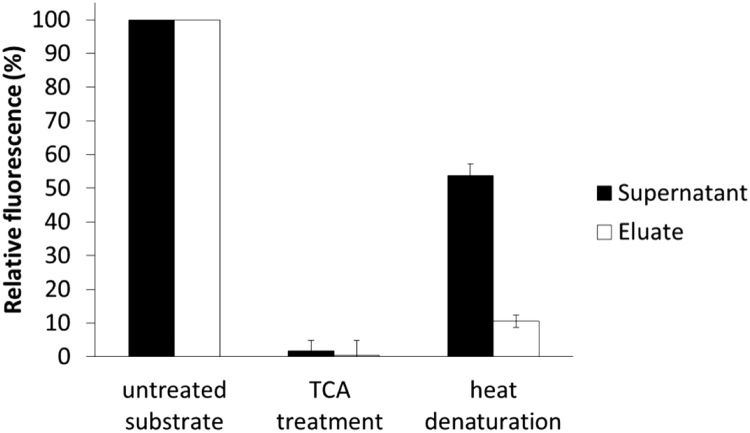
Effect of trichloroacetic acid (TCA) and heat-denaturation on substrate binding to Ni-NTA coated magnetic beads. To test the possible effects of two methods widely used for stopping enzymatic reactions on recombinant substrate properties, the pre-treatment of the His_6_-MBP-VSQNY↓PIVQ-mApple protein by TCA and by heat was performed. Eluate fractions contain the bead-attached molecules, while supernatant fractions contain the molecules remained in the solution. Relative fluorescence values were expressed as fluorescence relative to that obtained for the untreated substrate. Error bars represent SD (n = 2).

Data presented in this article provide information on characteristics of the His_6_-MBP-VSQNY↓PIVQ-mApple recombinant protein substrate (His_6_, hexahistidine tag; MBP, maltose binding protein; VSQNY↓PIVQ, arrow indicates cleavage position within the sequence of the matrix and capsid cleavage site of human immunodeficiency virus type 1 protease). This recombinant protein have been developed for a high throughput-compatible fluorescent protease assay [1], investigated properties (shown in Fig. 1, Fig. 2, Fig. 3, Fig. 4) of this substrate are important determinants of its usability.

## 2 Experimental design, materials and methods

All materials were purchased from Sigma-Aldrich (St Louis, MO, USA) unless otherwise indicated.

### 2.1 Substrate digestion by trypsin

Digestion of purified His_6_-MBP-VSQNY↓PIVQ-mApple recombinant substrate by trypsin was performed by incubating the reaction mixture in 0.1 M Tris buffer (1 mM CaCl_2_, 0.05% Tween 20, pH 8.0) at 37 °C for different incubation times. Reactions were stopped by the addition of loading buffer (6 × loading buffer, containing 12% SDS and 100 mM β-mercaptoethanol) to reaction mixtures followed by heat-treatment (incubation at 95 °C for 10 min). Separation of the substrates and cleavage products was carried out by denaturing SDS-PAGE using 14% polyacrylamide gel. The electrophoresis was followed by washing the gels with distilled water for 60 min at room temperature to renature the proteins. The fluorescent proteins in the unstained gels were detected by UV imaging using AlphaImager gel documentation system (ProteinSimple). Densitometry of gels was performed by using GelAnalyzer 2010a program (www.gelanalyzer.com).

Preparation of trypsin was performed based on the methods described previously [2], [3], [4].

### 2.2 Analysis of magnetic bead concentration-dependence of substrate binding

His_6_-MBP-VSQNY↓PIVQ-mApple recombinant substrate was incubated with Ni-NTA coated magnetic beads (Qiagen) at 37 °C for 20 min. Magnetic bead suspensions were added to the purified substrate solution in different concentrations, ranging from 0–25% (v/v). Magnetic beads were suspended in cleavage buffer (50 mM sodium acetate, 300 mM NaCl, 0.05% Tween 20, pH 7.0). The final volume of the solutions was set to 70 μl. After incubation the solutions were placed onto DynamagTM-2 magnetic particle concentrator (Thermo Fischer Scientific, Invitrogen) and the supernatants were removed from the beads (supernatant fractions). To prepare eluate fractions the bead-attached molecules were eluted from the beads by incubation in 70 μl elution buffer (100 mM EDTA, 0.05% Tween 20, pH 8.0) for 10 min at room temperature. The fluorescence of both the supernatant and eluate fractions were measured by using Biotek Synergy2 multimode plate reader at 590/35 nm excitation and 645/40 nm emission wavelengths.

### 2.3 Analysis of time-dependence of substrate binding

His_6_-MBP-VSQNY↓PIVQ-mApple recombinant substrate was incubated with Ni-NTA coated magnetic beads (Qiagen) at 37 °C for different times. Concentration of the applied magnetic bead suspension was set to be 5% (v/v), according to the manufacturer's protocol. The magnetic beads were suspended in cleavage buffer. After the incubation, the solutions were removed from the beads (supernatant fractions), and to prepare eluate fractions the bead-attached molecules were eluted from the beads by incubation in 70 μl elution buffer for 10 min at room temperature. The fluorescence of both supernatant and eluate fractions were measured by using Biotek Synergy2 multimode plate reader at 590/35 nm excitation and 645/40 nm emission wavelengths, followed by denaturing SDS-PAGE analysis of the samples using 14% polyacrylamide gel. To renature the proteins, after electrophoresis the SDS was washed out by rinsing the gels in distilled water for 60 minutes at room temperature. The fluorescent proteins in the unstained gels were detected by UV imaging using AlphaImager gel documentation system (ProteinSimple).

### 2.4 Effect of TCA- or heat-treatment on substrate binding

The His_6_-MBP-VSQNY↓PIVQ-mApple recombinant substrate was dissolved in cleavage buffer. In the case of TCA treatment, the TCA was added to the substrate solution in 5% (v/v) final concentration, and after centrifugation the precipitate was dissolved in cleavage buffer. In the case of heat-treatment, substrate solution was incubated at 95 °C for 10 min, followed by centrifugation and analysis of the soluble fraction of the sample. Both treatments were followed by incubation of the samples with 5% (v/v) magnetic bead suspension at 37 °C for 20 min, then the solutions were removed from the beads (supernatant fractions), and to prepare eluate fractions the bead-attached molecules were eluted by incubation of the beads in 70 μl elution buffer for 10 min at room temperature. The fluorescence of both supernatant and eluate fractions were measured by using Biotek Synergy2 multimode plate reader at 590/35 nm excitation and 645/40 nm emission wavelengths.

## References

[1] Bozóki B., Gazda L., Tóth F., Miczi M., Mótyán J.A., Tőzsér J. (2018). A recombinant fusion protein-based, fluorescent protease assay for high throughput-compatible substrate screening. Anal. Biochem..

[2] Sahin-Tóth M. (2000). Human cationic trypsinogen. Role of Asn-21 in zymogen activation and implications in hereditary pancreatitis. J Biol. Chem..

[3] Szabó A., Héja D., Szakács D., Zboray K., Kékesi K.A., Radisky E.S., Sahin-Tóth M., Pál G. (2011). High affinity small protein inhibitors of human chymotrypsin C (CTRC) selected by phage display reveal unusual preference for P4' acidic residues. J Biol. Chem..

[4] Szabó A., Salameh M.A., Ludwig M., Radisky E.S., Sahin-Tóth M. (2014). Tyrosine sulfation of human trypsin steers S2' subsite selectivity towards basic amino acids. PLoS One.

